# Together stronger: Intracolonial genetic variability occurrence in *Pocillopora* corals suggests potential benefits

**DOI:** 10.1002/ece3.5807

**Published:** 2020-06-05

**Authors:** Nicolas Oury, Pauline Gélin, Hélène Magalon

**Affiliations:** ^1^ UMR ENTROPIE (Université de La Réunion, IRD, CNRS) Université de La Réunion St Denis, La Réunion France; ^2^ Laboratoire d'Excellence CORAIL Perpignan France

**Keywords:** chimerism, intracolonial genetic variability, microsatellite, mosaicism, *Pocillopora*, scleractinian

## Abstract

We investigated the occurrence of intracolonial genetic variability (IGV) in *Pocillopora* corals in the southwestern Indian Ocean. Ninety‐six colonies were threefold‐sampled from three sites in Reunion Island. Nubbins were genotyped using 13 microsatellite loci, and their multilocus genotypes compared. Over 50% of the colonies presented at least two different genotypes among their three nubbins, and IGV was found abundant in all sites (from 36.7% to 58.1%). To define the threshold distinguishing mosaicism from chimerism, we developed a new method based on different evolution models by computing the number of different alleles for the infinite allele model (IAM) and the Bruvo's distance for the stepwise mutation model (SMM). Colonies were considered as chimeras if their nubbins differed from more than four alleles and if the pairwise Bruvo's distance was higher than 0.12. Thus 80% of the IGV colonies were mosaics and 20% chimeras (representing almost 10% of the total sampling). IGV seems widespread in scleractinians and beyond the disabilities of this phenomenon reported in several studies, it should also bring benefits. Next steps are to identify these benefits and to understand processes leading to IGV, as well as factors influencing them.

## Graphical abstract

We investigated the occurrence of intracolonial genetic variability (IGV) in *Pocillopora* corals in the southwestern Indian Ocean. We then developed a new method based on different evolution models to identify mosaic and chimeric colonies. Over 50% of the colonies presented at least two different genotypes, and the majority (80%) were mosaics. This suggests that IGV should be beneficial for genetically heterogeneous colonies.

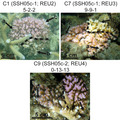

## 1 INTRODUCTION

Since the publication and the scientific recognition of the synthetic theory of evolution (Huxley, 1942), natural selection (i.e., the preservation of beneficial individual differences or variations and the disappearance of those that are deleterious in a given environment; Darwin, 1859) is recognized as the main engine of evolution. This natural selection acts on the individual, the latter being traditionally defined by the simultaneous and invariable presence of physiological unity and autonomy, genetic uniqueness, and genetic homogeneity (Santelices, 1999). However, this definition of the individual is disputable (see Pineda‐Krch & Lehtila, 2004) and many examples challenge it, such as intra‐organismal genetic heterogeneity (IGH; i.e., the presence of more than one genotype in a single organism; Rinkevich, 2001; Rinkevich & Weissman, 1987).

Usually, two kinds of IGH are distinguished, depending on the mechanism of formation: mosaicism and chimerism (Pineda‐Krch & Lehtila, 2004; Santelices, 1999). Mosaicism refers to organisms that are subject to intra‐organismal genetic modifications [e.g., somatic mutations, mitotic recombination, mitotic gene conversion (Otto & Hastings, 1998; Youssoufian & Pyeritz, 2002), or gene duplications (Santelices, 1999)], while chimerism designates a single organism resulting from the fusion or exchange of genetically distinct parts from different organisms (Rinkevich & Weissman, 1987). The most common mechanism leading to chimerism is the fusion of two organisms at a juvenile stage and then their mutual development (Barki, Gateño, Graur, & Rinkevich, 2002; Frank, Oren, Loya, & Rinkevich, 1997; Rinkevich & Weissman, 1987; Sommerfeldt, Bishop, & Wood, 2003). However, fusion at an adult stage remains possible (Sommerfeldt et al., 2003). Mosaicism and chimerism are also distinguished according to the degree of genetic differentiation among the genotypes present in a single organism (Schweinsberg, Weiss, Striewski, Tollrian, & Lampert, 2015). Indeed, mosaicism generally leads to small genetic variability among the different genotypes constituting the mosaic (only few nucleotides are added, modified or moved during a mutation event, resulting in one, sometimes two, different alleles; Schweinsberg et al., 2015). Resulting from the fusion of organisms, the chimera should probably show more genetic variability among its different genotypes (Santelices, 2004; Schweinsberg et al., 2015). Chimerism seems rarer than mosaicism, partly due to the specificities of its mechanisms of formation (Pineda‐Krch & Lehtila, 2004; Rinkevich, 2004; Santelices, 2004). The successful formation of a chimera needs (a) the physical contact of two organisms at a juvenile stage allowing fusion, (b) restrictive suitable environmental conditions, and (c) overriding the allorecognition barrier (Rinkevich, 2004; Santelices, 2004). Thus, while mosaicism seems possible in all animal and plant taxa, chimerism occurs only in some, including marine benthic organisms with early planktonic stages (Santelices, 2004). Some of these organisms, like scleractinian corals, usually adopt strategies of synchronous releases of propagules to increase their fitness (Harrison, 2011; Richmond & Hunter, 1990). These strategies induce aggregations of propagules, multiplying opportunities of contact, and fusion (Barki et al., 2002; Jiang, Lei, Liu, & Huang, 2015). In some species, the planktonic propagules tend to gregariously recruit on some substrates, increasing the probability of fusion among organisms (Puill‐Stephan, Oppen, Pichavant‐Rafini, & Willis, 2012).

IGH has long been seen as a potential threat for solitary organisms as it could lead to antagonistic interactions among different genotypes, reducing cooperation, and intercellular exchanges among them (as for the formation of tumors and autoimmune diseases; Amar, Chadwick, & Rinkevich, 2008; Chadwick‐Furman & Weissman, 1995; Pineda‐Krch & Lehtila, 2004). In some extreme cases, IGH would cause the death of one or more genotypes, or even of the whole organism. Until recently, viable IGH was considered as exceptional (Santelices, 2004). However, it seems to present some benefits (reviewed in Ben‐Shlomo, 2017), such as increasing phenotypic plasticity (Medina, Flores, González, & Santelices, 2015) and improving growth (Grosberg, 1988; Maier, Buckenmaier, Tollrian, & Nürnberger, 2012), competitive abilities (Ballarin, Du Pasquier, Rinkevich, & Kurtz, 2015; Forsman, Page, Toonen, & Vaughan, 2015), survival (Maier et al., 2012), and/or fitness of the organism (Folse & Roughgarden, 2012; Grosberg, 1988). This is particularly true in colonial organisms (Maier et al., 2012; Pineda‐Krch & Lehtila, 2004) where intracolonial genetic variability (IGV; i.e., the presence of more than one genotype in a single colony; Schweinsberg et al., 2015), instead of compromising the cooperation among the physiological units composing the colony, was shown viable in different marine animal taxa: tunicates (e.g., Ben‐Shlomo, Motro, Paz, & Rinkevich, 2007; Pancer, Gershon, & Rinkevich, 1995; Rinkevich & Yankelevich, 2004), sponges (Maldonado, 1998), bryozoans (Hughes, Ayre, & Connell, 1992), hydrozoans (Dubé, Planes, Zhou, Berteaux‐Lecellier, & Boissin, 2017; Lakkis, Dellaporta, & Buss, 2008; Schweinsberg, Tollrian, & Lampert, 2017), alcyonaceans (Barki et al., 2002), or scleractinians (e.g., Rinkevich, Shaish, Douek, & Ben‐Shlomo, 2016; Schweinsberg et al., 2015; Work et al., 2011). Besides being viable in these taxa, IGV was also found in high prevalence, notably among scleractinian corals (up to 50%; Puill‐Stephan et al., 2012; Schweinsberg et al., 2015). In addition, mosaicism has been reported as the main process leading to IGV (e.g., 90% of the IGV colonies were mosaic in Schweinsberg et al., 2015). Such high IGV proportions suggest that it might be beneficial for genetically heterogeneous colonies and of potential interest for scleractinian corals, in the context of declining coral reefs (Wilkinson, 2008). Therefore, it appears mandatory to better understand these advantages and the processes leading to IGV and to assess its occurrence in coral species and populations.

Among scleractinians, this study focused on *Pocillopora* corals from the southwestern Indian Ocean. More precisely, we focused on *Pocillopora damicornis* type *β* (or *Pocillopora acuta *sensu Schmidt‐Roach, Miller, Lundgren, & Andreakis, 2014), which was recently demonstrated as a species complex (see Gélin, Pirog, Fauvelot, & Magalon, 2018 and Gélin, Postaire, Fauvelot, & Magalon, 2017 for more details). Besides, in the southwestern Indian Ocean, *P. damicornis* type *β* species complex comprises two secondary species hypotheses (SSHs), SSH05c and SSH05d, that are exclusively found in this region, sometimes in sympatry. Moreover, *Pocillopora* SSH05c shows a deeper partitioning in two diverging, but sympatric, genetic groups (Gélin, Fauvelot, et al., 2017; Gélin, Pirog, et al., 2018). For now, only few studies investigated IGV in *Pocillopora* corals. Briefly, IGV was first identified in *P. damicornis sensu lato* colonies (the species complex was not highlighted yet) from Hawaii (Hidaka, 1985) and Okinawa (Japan; Hidaka, Yurugi, Sunagawa, & Kinzie, 1997) with histocompatibility and allorecognition studies. More recently, using microsatellites, IGV was involved in *P. damicornis sensu lato* larvae from Thailand and Philippines (Rinkevich et al., 2016) and in *Pocillopora* spp. colonies (a mix of *P. damicornis sensu stricto*, *P. acuta*, and unidentified *Pocillopora* colonies) from Lizard Island (Australia; Schweinsberg et al., 2015). Here, focusing on *Pocillopora* species from the southwestern Indian Ocean (Reunion Island), we aimed to evaluate the occurrence of IGV and consequently each process leading to it (i.e., chimerism and mosaicism). Besides, we aimed to test whether its occurrence was linked to colony density, assuming that higher density should increase the contact probability between entities (larvae or recruits) and thus the probability to produce chimeras. For this, in each of the three sites chosen for their contrasting colony densities, 32 colonies were haphazardly chosen (i.e., while snorkelling, without randomly predefined sampling points) and threefold‐sampled, each nubbin being genotyped using 13 specific microsatellite loci. As colony macromorphology is not a discriminant character in *Pocillopora* genus (Gélin, Postaire, et al., 2017; Pinzón et al., 2013; Schmidt‐Roach et al., 2014), species identification of the colonies was verified a posteriori using assignment methods. To evaluate the proportion of IGV, the multilocus genotypes (MLGs) were compared among intracolonial nubbins using two differentiation indices, each based on a different evolution model [number of different alleles for the infinite allele model (IAM) and Bruvo's distance for the stepwise mutation model (SMM)]. As some microsatellite loci can mutate without following the SMM (Di Rienzo et al., 1994), using both evolution models seems more representative of the mutation mechanisms occurring in microsatellites. Then the proportions of mosaicism and chimerism were calculated using a new method to define the threshold between both processes. These results should help understanding IGV and the processes leading to it in corals, as well as the potential benefits of having multiple genotypes in a context of declining coral reefs.

## 2 MATERIALS AND METHODS

### 2.1 Sampling design

**Figure 1:**
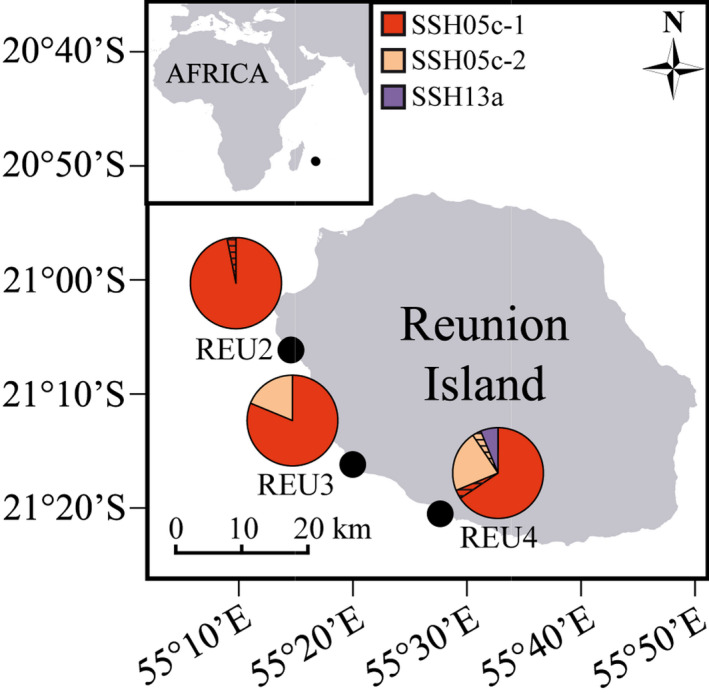
Sampling sites of *Pocillopora* colonies in Reunion Island (represented by the black circles). For each site (*N* = 32 colonies), the species and cluster distribution are given. The hatched parts correspond to colonies removed as no comparison among nubbin genotypes was possible (no locus in common)

Adult colonies presenting *Pocillopora damicornis*‐like *corallum* macromorphology were sampled on three sites of the west coast of Reunion Island (southwestern Indian Ocean, 700 km east of Madagascar; Figure 1) in March 2017. These sites, formerly sampled in a previous study focusing on clonal propagation (see Gélin, Fauvelot, et al., 2017, for a description of each site, the site code being consistent from one study to another), were chosen for their contrasted environmental conditions and differences in *Pocillopora* densities: from north to south and from denser to less dense, REU2 (Trou d'Eau; 21°06′08.86″S, 55°14′34.08″E), REU3 (Étang Salé; 21°16′11.28″S, 55°19′59.09″E), and REU4 (Saint‐Pierre; 21°20′31.02″S, 55°27′39.67″E).

On each site, 32 colonies were haphazardly chosen and threefold‐sampled (+photographed) by cutting three nubbins (branch tip of <1 cm), using pliers. To enhance the probability of discovering multiple genotypes in a single colony, the nubbins within a colony were collected by maximizing the distance among them. Adopting a geometric approach, it means that the three nubbins were taken from the vertices of a virtual triangle modeled on the surface of the colony with the maximum area possible. In case of bicolor colonies, the respective color of each nubbin was noted. Each collected nubbin was isolated into a numbered zip‐lock bag on the field, then fixed in 90% ethanol at laboratory and stored at room temperature.

### 2.2 Genotyping and *Pocillopora* species identification

From small pieces of the collected nubbins (total volume of ca. 3 mm^3^), DNA was extracted using DNeasy Blood & Tissue kit (Qiagen™) following the manufacturer's protocol. Genotyping and post‐PCR multiplexing were performed with 13 microsatellite loci, as in Gélin, Postaire, et al. (2017; Table S1). Loci showing ambiguous peak profiles (e.g., faint peaks or more than two peaks) were processed again in simplex and, if remaining ambiguous, designated as missing data. The percentage of missing data was estimated for each locus, and samples with no readable locus were not kept for further analysis.

As colonies were sampled based on their macromorphology, a nondiscriminant character in this genus (e.g., colonies showing *P. damicornis*‐like macromorphology could be members of *Pocillopora verrucosa* or *P. damicornis* type *β* species complexes; Gélin, Postaire, et al., 2017; Pinzón et al., 2013; Schmidt‐Roach et al., 2014), identification of *Pocillopora* species was performed a posteriori of the sampling. Besides, in the southwestern Indian Ocean, *P. damicornis* type *β* species complex comprises two secondary species hypotheses (SSHs), SSH05c and SSH05d, that are exclusively found in this region, sometimes in sympatry (see Gélin, Pirog, et al., 2018; Gélin, Postaire, et al., 2017). Moreover, *Pocillopora* SSH05c shows a deeper partitioning in two diverging, but sympatric, genetic groups, hereafter referred as clusters [Clusters 1 and 2 in Gélin, Fauvelot, et al. (2017) and corresponding respectively to Clusters 2 and 3 in Gélin, Pirog, et al. (2018)]. First, performing Bayesian assignment tests with Structure 2.3.4 (Pritchard, Stephens, & Donnelly, 2000) as in Gélin, Fauvelot, Bigot, Baly, & Magalon (2018), colonies were assigned to one SSH (assignment probability ≥0.75). Then, for colonies assigned to *Pocillopora* SSH05c, to identify SSH05c clusters, these colonies were added to the dataset of Gélin, Pirog, et al. (2018; i.e., the truncated dataset containing one representative per MLG and population). Structure was then run as in Gélin, Pirog, et al. (2018), and these colonies were assigned (assignment probability ≥0.75) to one of the two SSH05c clusters [named hereafter to ease reading SSH05c‐1 and SSH05c‐2 instead of SSH05c Cluster 1 and 2 sensu Gélin, Fauvelot, et al. (2017)]. Finally, MLGs of these SSH05c colonies were compared to those of the colonies from Gélin, Fauvelot, et al. (2017) as, studying asexual reproduction of *Pocillopora* SSH05c in the same sites as the present study, the authors found some clones that were much more frequent than others, especially at site REU2 (up to 81%). The software GenClone 2.0 (Arnaud‐Haond & Belkhir, 2007) was used and only MLGs without missing data were compared.

### 2.3 Intracolonial genetic variability analysis

To identify IGV, all possible pairwise comparisons between MLGs from nubbins within the same colony were made. To deal with missing data, for each MLG involved in the comparison, only loci that correctly amplified were kept. Thus we noted *N_L_*, the number of comparable loci between two intracolonial nubbins. Then, for each comparison between two MLGs, we calculated, using basic R 3.3.1 functions (R Core Team, 2016), *N_A_*, the number of different alleles and *D*, the Bruvo's distance (Bruvo, Michiels, D'Souza, & Schulenburg, 2004), computed as $D=\frac{\sum_{i=1}^{l}1-2^{-\left|x\right|}}{2l}$, where *l* is the total number of loci and *x*, the number of different mutation steps between two alleles. Thus, while *N_A_* is rather based on the infinite allele model (IAM; Kimura & Crow, 1964), *D* is based on the stepwise mutation model (SMM; Kimura & Ohta, 1978). Both indices allow a comparison of two MLGs according to both mutation models and should provide a better estimate of the differentiation between MLGs.

#### 2.3.1 Invariable/variable colonies

When *N_A_* ≥ 1 and *D* > 0 for at least one comparison among intracolonial MLGs, this colony was considered as *variable* (i.e., presenting IGV). On the contrary, colonies for which all sampled nubbins shared the same MLG (i.e., *N_A_* = *D* = 0, for each intracolonial comparison) were considered as invariable. However, this last consideration largely depends on the number of comparable loci between two intracolonial nubbins, *N_L_*: when *N_L_* is low (due to missing data), some loci were not compared, limiting the detection of variable colonies. Thus, we distinguished the colonies *invariable* (*N_L_* sufficiently high in all intracolonial comparisons to confidently consider that the nubbins share the same MLG) and the colonies invariable but *possibly variable* (*N_L_* too low to affirm with certainty that colonies are not *variable*). To distinguish these two categories, a threshold of *N_L_* was defined by plotting its distribution for all comparisons within invariable colonies (Figure S1). We also estimated the probability of detecting a colony as invariable, while it is actually *variable* for a given *N_L_* (i.e., a kind of false‐negative probability). For that, we considered all nubbin pairs that (a) had no missing data (*N_L_* = 12 loci) and (b) were variable (number of different alleles, *N_A_* > 0 and Bruvo's distance, *D* > 0). It represented a total of 17 pairs (original dataset; see Results). Then, for each value of *N_L_* (varying from 1 to 11), we removed all possible combinations of *L* loci to reach a given *N_L_* (*N_L_* = 12 − *L* loci). From these 11 new datasets, the “false negative” probability was then estimated as the number of pairs that became invariable after removing *L* loci over the total number of pairs of each new dataset (i.e., $C_{12}^{L}\times 17$; Figure S1). Looking both at the distribution and the “false negative” probability, the threshold was defined at *N_L_* = 9 loci (representing the first antimode of the distribution and a “false negative” probability of 16.7%; Figure S1). When *N_L_* < 9 loci, the probability of detecting a colony as invariable, while it is actually *variable* was superior to 20%. Afterward, colonies were *invariable* if *N_A_* = *D* = 0 and *N_L_* ≥ 9, for each intracolonial comparison, and *possibly variable* if *N_A_* = *D* = 0, for each intracolonial comparison, but *N_L_* < 9 in at least one comparison.

If colonies were found *variable* with only one locus differing among the genotypes of the nubbins, this locus was reamplified for the differing genotypes to exclude genotyping errors.

#### 2.3.2 Mosaic/chimeric colonies

To distinguish chimeric from mosaic colonies among those previously identified as *variable*, a genetic differentiation threshold beyond which colonies were considered as chimeras was also defined for each genetic distance (noted *N_A_*_ CHI/MOS_ and *D*_ CHI/MOS_, respectively). This threshold assumes that mosaic genotypes should only differ from a few mutations (i.e., *N_A_* and *D* are low), while chimeric genotypes should exhibit higher *N_A_* and *D*. All nubbin genotypes without missing data were compared by pair and the distributions of *N_A_* and *D* were plotted. For a given species, these distributions are expected to be bimodal: the first mode, in low values, should correspond to differences due to somatic mutations, while the second mode, in higher values, should correspond to chimerism. The genetic differentiation threshold distinguishing chimerism from mosaicism (*N_A_*_ CHI/MOS_ or *D*_ CHI/MOS_) would therefore be the first antimode of the distribution.

Afterward, colonies previously identified as *variable* and for which *N_A_* > *N_A_*_ CHI/MOS_ and *D* > *D*_ CHI/MOS_ for at least one intracolonial comparison were considered as *chimeric*. The others were mosaic (i.e., *N_A_* ≥ 1 and *D* > 0 for at least one comparison, but *N_A_* ≤ *N_A_*_ CHI/MOS_ or *D* ≤ *D*_ CHI/MOS_ for all comparisons). Thus, nubbins with MLGs for which *N_A_* = *N_A_*_ CHI/MOS_ or *D* = *D*_ CHI/MOS_ were considered as mosaic. As for the invariable colonies (as determined in the previous section), two categories of mosaic colonies were distinguished, depending on *N_L_*: (a) colonies *mosaic* (*N_L_* sufficiently high to consider the colonies as mosaic) and (b) colonies mosaic but *possibly chimeric* (*N_L_* too low to affirm with certainty that colonies are not *chimeric*). The same *N_L_* threshold as previously (i.e., distinguishing *invariable* from *possibly variable* colonies: *N_L_* = 9) was considered for parsimony. Noteworthy, some colonies could be both *chimeric* and *mosaic* if *N_A_* > *N_A_*_ CHI/MOS_ and *D* > *D*_ CHI/MOS_ for two nubbins and the third differs from the two previous such that 0 < *N_A_* ≤ *N_A_*_ CHI/MOS_ or 0 < *D* ≤ *D*_ CHI/MOS_.

For each SSH and each cluster identified a posteriori, the proportions of colonies belonging to each category of genetic variability (i.e., *invariable*, *possibly variable*, *mosaic*, *possibly chimeric*, and *chimeric*) were calculated per site and on all colonies. The distributions of the invariable (*invariable* + *possibly variable*), mosaic (*mosaic* + *possibly chimeric*), and *chimeric* colonies were compared among sites and among SSHs and clusters, using Fisher's exact tests with R 3.3.1 (R Core Team, 2016).

## 3 RESULTS

### 3.1 Genotyping and *Pocillopora* species identification

Among the 96 sampled colonies, all nubbins from the same colony were assigned to the same SSH and then to the same cluster. Thus two colonies (in REU4) were assigned to *Pocillopora* SSH13a (*P. verrucosa *sensu Schmidt‐Roach et al., 2014; Figure 1) and 94 to *Pocillopora* SSH05c (REU2: 32; REU3: 32; REU4: 30), of which 80 were further assigned to SSH05c‐1 (REU2: 32; REU3: 26; REU4: 22) and 14 to SSH05c‐2 (REU3: 6; REU4: 8). Among the 115 nubbins presenting no missing data in their MLG at 13 loci (57 colonies; only belonging to SSH05c), 71 (61.7%; 42 colonies) presented an MLG already sampled in Gélin, Fauvelot, et al. (2017). In particular, five of the ten most‐represented MLGs in this previous study (MLG01, MLG02, MLG03, MLG06, and MLG08) were retrieved in 54 nubbins (33 colonies), including the most frequent one [MLG01, found in REU2 (25 nubbins; 16 colonies) and REU3 (five nubbins; four colonies)], which was previously found overrepresented in REU2 (81%; Gélin, Fauvelot, et al., 2017). Then, locus Pd4 was no longer used for further analyses due to potential genotyping errors (three‐peak electrophoregrams). Proportions of missing data per locus (all colonies considered) varied from 11.8% for Pd3‐004 to 41.7% for Pd3‐009 for the 12 remaining loci (Table S1). Two colonies from SSH05c‐1 (REU2: 1 and REU4: 1) and one from SSH05c‐2 (REU4) were removed as no comparison between nubbin pairs was possible (no locus in common). The final dataset thus comprised 93 *Pocillopora* colonies (Figure 1): 91 SSH05c colonies (REU2: 31; REU3: 32; REU4: 28) and two SSH13a colonies (REU4).

### 3.2 Intracolonial genetic variability analysis

#### 3.2.1 Invariable/variable colonies

**Figure 2:**
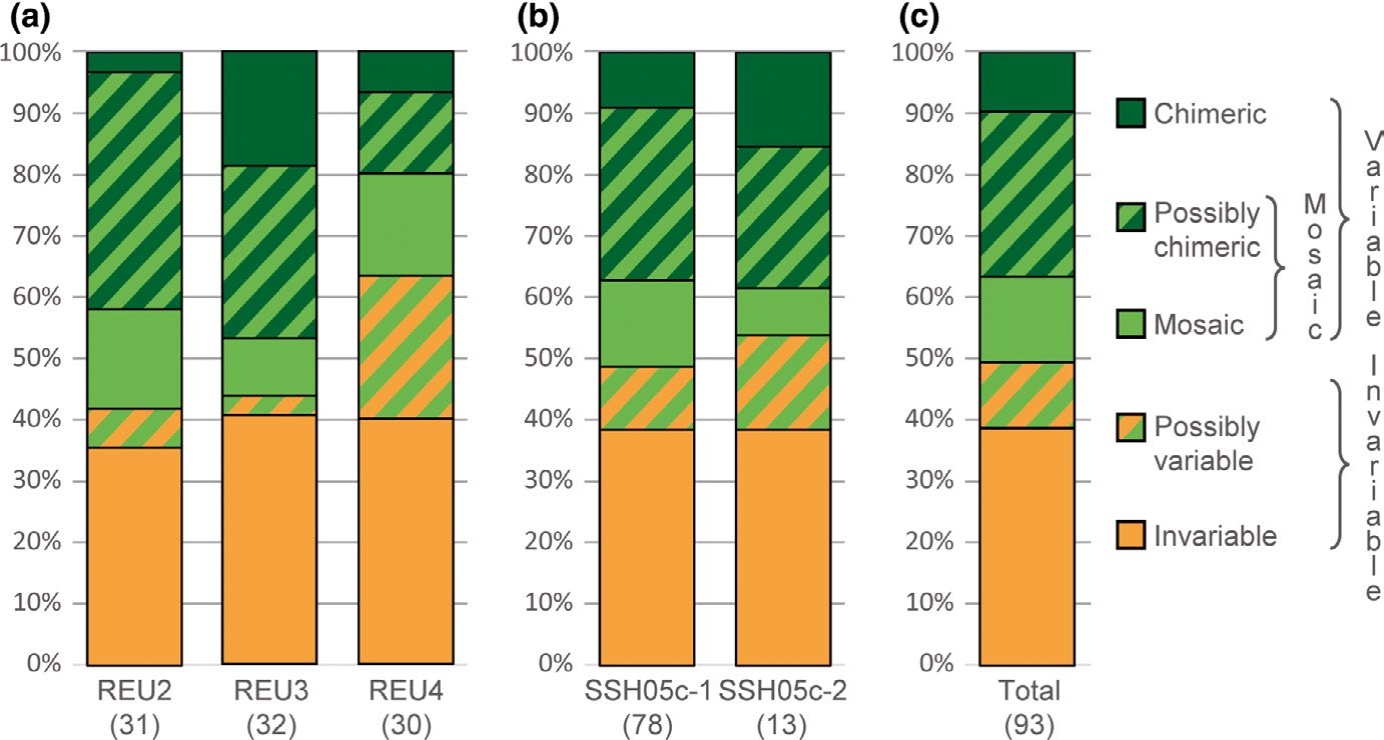
Proportions of the categories of genetic variability (a) per site, (b) per SSH05c cluster (SSH13a was not represented as only two colonies from REU4 were sampled), and (c) overall colonies (number of colonies in parentheses). The hatched parts correspond to colonies for which at least one intracolonial comparison was done with less than nine loci (*N_L_* < 9). Distributions are not significantly different among sites (Fisher's exact test; *p* = .099) nor between clusters (Fisher's exact test; *p* = .626)

Of the 93 colonies (78 SSH05c‐1, 13 SSH05c‐2, and 2 SSH13a), 47 (50.5%) were *variable* (i.e., displaying more than one genotype; SSH05c‐1: 51.3%; SSH05c‐2: 46.2%; SSH13a: 50.0%; Figures 2 and S2), with *N_A_* varying from 0 to 13 alleles and *D* varying from 0 to 0.37. Among sites, variable colonies represented from 36.7 (REU4) to 58.1% (REU2) of the colonies (Figures 2 and S2). Concerning the 46 remaining colonies, 36 were *invariable* (SSH05c‐1: 30/38; SSH05c‐2: 5/7; SSH13a: 1/1) and 10 were *possibly variable* (i.e., *N_L_* < 9 in at least one intracolonial comparison; Figures 2 and S2).

#### 3.2.2 Mosaic/chimeric colonies

**Figure 3:**
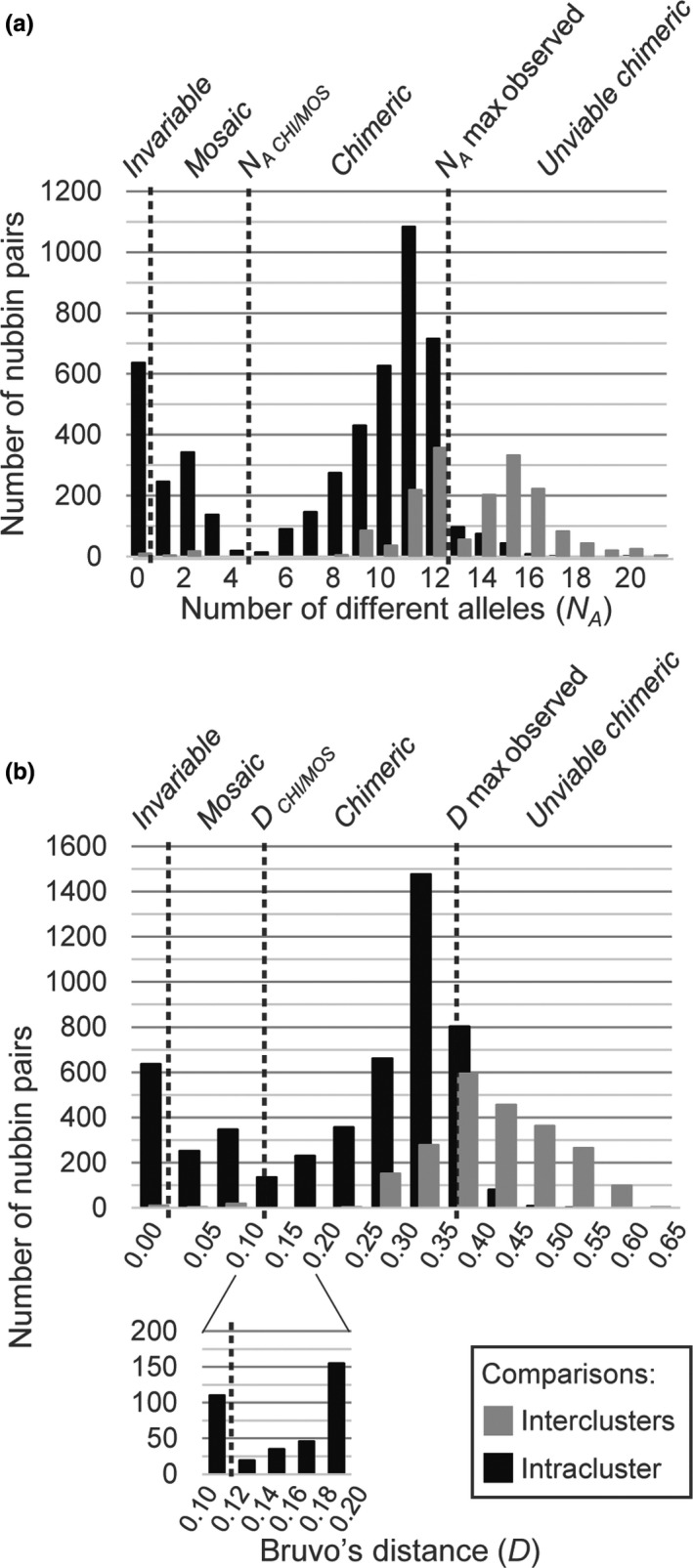
Thresholds between mosaicism and chimerism. (a) Distribution of the number of different alleles (*N_A_*) between two multilocus genotypes (MLGs) and (b) distribution of the Bruvo's distance (*D*; Bruvo et al., 2004) between two MLGs. Only MLGs without missing data were compared by pair (*N* = 6,670 paired comparisons, including 4,987 intracluster, and 1,683 intercluster comparisons). The categories of genetic variability are indicated above each chart. *N_A_*_ CHI/MOS_ and *D*_ CHI/MOS_ are the genetic differentiation thresholds between mosaicism and chimerism

Considering all loci except Pd4, 116 nubbins (SSH05c‐1: 99; SSH05c‐2: 17; SSH13a: 0) presented a MLG without missing data. Thus, these 116 MLGs (only from SSH05c) were compared by pair to define the thresholds between chimerism and mosaicism (*N_A_*_ CHI/MOS_ and *D*_ CHI/MOS_). Intracluster and intercluster comparisons were distinguished. From the resulting 4,987 intracluster comparisons, both *N_A_* and *D* distributions showed two modes (*N_A_* = 2 and *N_A_* = 11; *D* ≈ 0.08 and *D* ≈ 0.33) and one antimode (between *N_A_* = 4 and *N_A_* = 5; *D* = 0.12; Figure 3). As explained previously, mosaicism should be centred on the lowest mode (near *N_A_* = 2 and *D* = 0.08) and chimerism on the second mode (near *N_A_* = 11 and *D* = 0.33). Assuming that, the thresholds distinguishing mosaicism and chimerism (*N_A_*_ CHI/MOS_ and *D*_ CHI/MOS_) were defined at the first antimode of each distribution: a colony was considered as *chimeric* when the MLGs of at least two nubbins differed such as *N_A_* > 4 and *D* > 0.12 [Figure 3; as an illustration, over 12 loci (24 alleles), a *D* = 0.125 could correspond (among other combinations) to two MLGs differing by four alleles, each differing by two mutation steps; a *D* = 0.120 could correspond (among other combinations) to two MLGs differing by (a) two alleles, each differing by one mutation step, along with two alleles, each differing by four mutation steps, or (b) two alleles, each differing by five mutation steps, along with one differing by four mutation steps]. Noteworthy, *N_A_* and *D* were higher for intercluster comparisons than for intracluster comparisons and thus formed a third mode in both distributions (*N_A_* = 15 and *D* ≈ 0.38) and a second antimode in the distribution of *N_A_* (near *N_A_* = 13; Figure 3). This latter could correspond to the gap distinguishing both SSH05c clusters, consolidating their existence (Gélin, Fauvelot, et al., 2017; Gélin, Pirog, et al., 2018). Additionally, some intracluster comparisons led to *N_A_* and *D* higher than the maximum values observed in intracolonial comparisons (i.e., *N_A_* = 13 and *D* = 0.37; Figure 3). We admitted that it could correspond to unviable chimerism (discussed later). To allow some inter‐SSH comparisons, we repeated the same analysis at 11 loci (removing PV2) so that SSH13a nubbins could be included, that is, comparing 120 nubbins without missing data (SSH05c‐1: 99; SSH05c‐2: 17; SSH13a: 4; Figure S3). For both distributions of *N_A_* and *D*, the same modes and antimodes were observed (slightly lower due to removal of a locus; Figure S3) for intra‐ and intercluster comparisons. Inter‐SSH comparisons (*N* = 464) were responsible for a fourth mode (*N_A_* = 19 and *D* ≈ 0.68; Figure S3), higher than the one due to intercluster comparisons.

**Figure 4:**
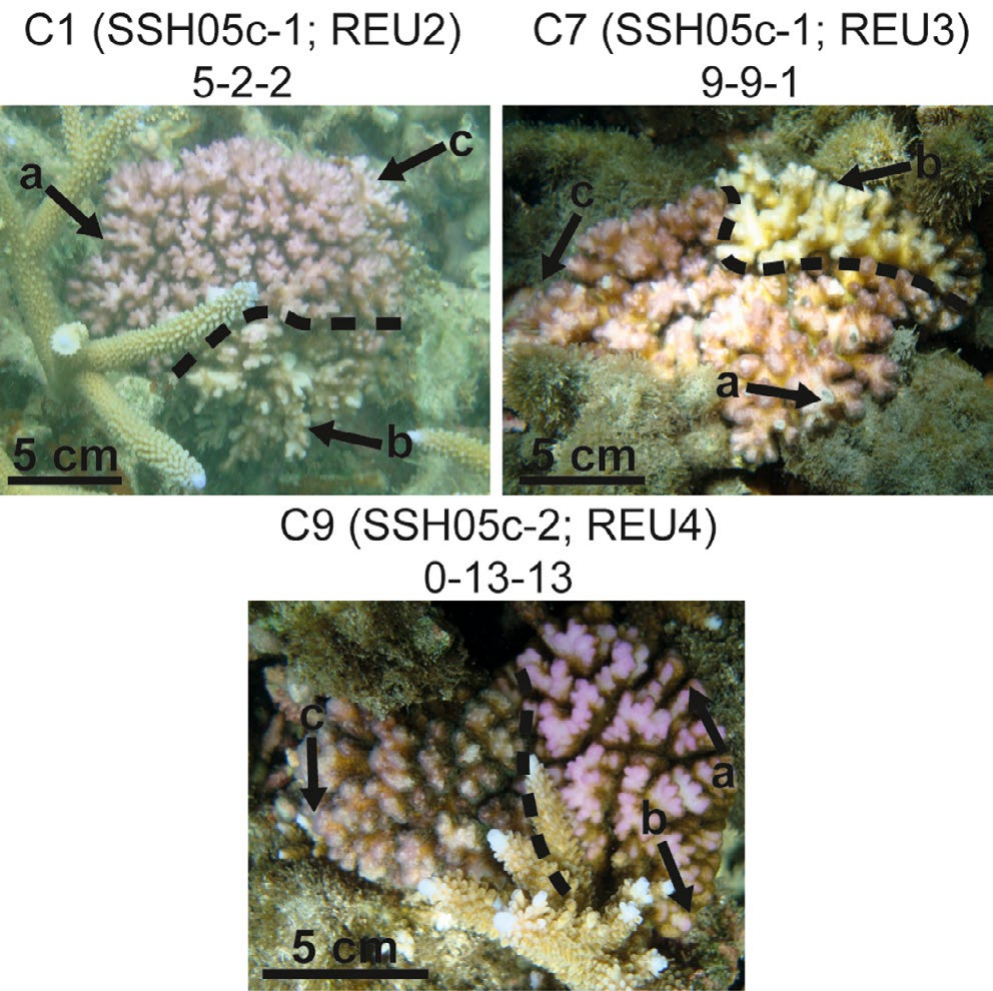
Pictures of the three bicolor *Pocillopora* SSH05c colonies. Colors are delimited with the dashed line and nubbin sampling spots (noted a, b, and c within each colony, referring to Table S2) are shown with the arrows. At the top of each photo, are indicated (1) the name of the colony (referring to Table S2), (2) the cluster and the site (in parentheses), and (3) the numbers of different alleles (*N_A_*) between two multilocus genotypes (MLGs) for the intracolonial comparisons of nubbins a‐b, b‐c and a‐c, respectively

Using the thresholds defined above, among the 47 variable colonies, 38 (80.9%) were mosaic (SSH05c‐1: 33/40; SSH05c‐2: 4/6; SSH13a: 1/1) of which six presented three distinct MLGs each, and thus nine colonies (19.1% of the variable colonies and 9.7% of all colonies) were *chimeric* (SSH05c‐1: 7/40; SSH05c‐2: 2/6; SSH13a: 0/1), among which seven were also mosaic. Mosaic colonies represented from 66.7 (REU3) to 94.4% (REU2) of the variable colonies per site and from 30.0 (REU4) to 54.8% (REU2) of all colonies per site (Figures 2 and S2). However, only 13 colonies (SSH05c‐1: 11/33; SSH05c‐2: 1/4; SSH13a: 1/1) were *mosaic*, the 25 others were *possibly chimeric* (i.e., *N_L_* < 9 in at least one intracolonial comparison; Figures 2 and S2). At least one chimera was found per site (REU2: 1; REU3: 6; REU4: 2). Thus proportions of chimeric colonies per site varied from 3.2 (REU2) to 18.8% (REU3; Figures 2 and S2). Considering the thresholds *N_A_*_ CHI/MOS_ = 4 and *D*_ CHI/MOS_ = 0.12, the two distances used were congruent, except for one colony of SSH05c‐1 considered as *chimeric* according to Bruvo's distance (*D* = 0.15) but *mosaic* according to the number of different alleles (*N_A_* = 4). Interestingly, six MLGs were shared among different variable colonies, including three that were shared among different chimeras (see Table S2). Besides three chimeras were bicolor (SSH05c‐1: 2; SSH05c‐2: 1; Figure 4): nubbins of the same color were less genetically different (*N_A_* ≤ 2 and *D* ≤ 0.05) than those of different colors (*N_A_*_ CHI/MOS_ < *N_A_* ≤ 13 and *D*_ CHI/MOS_ < *D* ≤ 0.37; Figure 4; Table S2).

Among sites, no significant difference in the distribution of the invariable (*invariable* + *possibly variable*), mosaic (*mosaic* + *possibly chimeric*), and *chimeric* colonies were found (Fisher's exact test; *p* = .099; Figure 2a). Additionally, no significant difference was found between SSH05c clusters (all sites pooled; Fisher's exact test; *p* = .626; Figure 2b). SSH13a was not compared with SSH05c as only two colonies were sampled.

## 4 DISCUSSION

This study of IGV highlighted the existence of the phenomenon in high rates in different populations of *Pocillopora* corals from the southwestern Indian Ocean (from 36.7% to 58.1%). Moreover, IGV was found in each species and cluster, in similar proportions (SSH05c‐1: 51.3%; SSH05c‐2: 46.2%; SSH13a: 50.0%). More than 80% of the variable colonies were mosaics, suggesting that mosaicism is the major process leading to IGV. However, some relatively high rates of chimerism were also found (about 10% of all colonies), implying that it should not be neglected. The proportions of the invariable, mosaic, and chimeric colonies were similar among the three sampled sites. Thus no effect of the colony density on the production of chimeras was detected. However, additional factors such as contrasting environmental conditions among the three sites or clonality could offset and hide the effect of colony density.

### 4.1 IGV: the production of “super corals?”

IGV has long been considered as rare and disabling for organisms [Pineda‐Krch & Lehtila, 2004; e.g., in the coral *Stylophora pistillata* (Amar et al., 2008) or in the ascidian *Botryllus schlosseri* (Chadwick‐Furman & Weissman, 1995)]. However, recent investigations demonstrated that genetic heterogeneity is widespread in plants (see Herrera, 2009, for a review) and in different marine animal taxa [e.g., in tunicates (Ben‐Shlomo et al., 2007; Rinkevich, 2005), sponges (Maldonado, 1998), or bryozoans (Hughes et al., 1992)], including in scleractinian corals (Barki et al., 2002; Ben‐Shlomo, Douek, & Rinkevich, 2001; Conlan, Humphrey, Severati, & Francis, 2018; Frank et al., 1997; Puill‐Stephan et al., 2012; Schweinsberg et al., 2015). For example, Puill‐Stephan et al. (2012) showed, using nine microsatellite loci, that 50% of recently settled juveniles of *Acropora millepora* presented more than one genotype, in experimental conditions. Moreover, Schweinsberg et al. (2015) obtained between 24% and 47% of genetically variable colonies in five scleractinian taxa: *Acropora florida*, *Acropora hyacinthus*, *Acropora sarmentosa*, *Pocillopora* spp., and *Porites australiensis*, using eight microsatellite loci per taxon. In this study, we also obtained a high proportion of genetically variable colonies in the *Pocillopora* genus (50.5%), using 12 microsatellite loci. This proportion is twofold higher than the one obtained by Schweinsberg et al. (2015) on *Pocillopora* spp. (23.8% for *N* = 42 colonies, including five colonies of *P. damicornis sensu stricto*, two colonies of *P. acuta*, and 35 unidentified colonies), with a very similar method. Moreover, the phenomenon was found in relatively similar proportions among the sampled species and clusters (SSH05c‐1: 51.3%; SSH05c‐2: 46.2%; SSH13a: 50.0%) and sites (from 36.7% to 58.1%), demonstrating how widespread IGV is.

The presence of more than one genotype in a single colony imbues both disadvantages and advantages for the colony. On one hand, it may lead to competition among the different genotypes that may be detrimental for the colony (Pineda‐Krch & Lehtila, 2004). On the other hand, it results in a higher genetic variability in the colony, but also in the population, as all genetic parts are theoretically able to reproduce (van Oppen, Souter, Howells, Heyward, & Berkelmans, 2011). This greater genetic variability provides several genotypes upon which selection processes may act, which could lead to differential selection among intracolonial genotypes (i.e., intra‐organismal selection; Otto & Orive, 1995). IGV also brings benefits for the colony growth (Maier et al., 2012; e.g., chimerism was reported as the major growth mechanism in the hydrozoan *Ectopleura larynx*; Chang, Orive, & Cartwright, 2018), its competitive ability (Ballarin et al., 2015; Forsman et al., 2015; Nicotra, 2019), its survival (Maier et al., 2012) and its fitness (Santelices, 2004), such benefits that might be of potential interest in the context of global changes and declining coral reefs. Indeed, while coral assisted evolution (i.e., enhance the ability of corals to tolerate stressful environments and accelerate recovery after acute impacts through genetic engineering; van Oppen, Oliver, Putnam, & Gates, 2015) is considered as a potential solution to face these changes, IGV might be the natural way to produce “super corals” (see Rinkevich, 2019). As it is commonly accepted that populations with greater genetic diversity will have higher evolutionary potential (i.e., greater ability to survive selection pressures; see Frankham, Bradshaw, & Brook, 2014), colonies presenting IGV should theoretically have a better evolutionary potential than invariable colonies. Indeed, presenting multiple genotypes should provide several basic units upon which selection may act. Yet, actual knowledge about IGV and its benefits are insufficient to accurately state on the ecological and evolutionary implications of the phenomenon.

### 4.2 Threshold between mosaicism and chimerism

In this study, as in others (e.g., Dubé et al., 2017; Puill‐Stephan et al., 2012; Schweinsberg et al., 2015), we assumed a threshold of genetic differentiation distinguishing mosaicism and chimerism. Considering only intracluster comparisons, this threshold was defined at the first antimode of the distributions of two MLG differentiation indices (number of different alleles and Bruvo's distance), each based on a different mutation model (IAM and SMM, respectively). Intercluster and inter‐SSH comparisons led to higher genetic differentiation indices. We assumed that such genetic distances could correspond to unviable chimerism as (a) no variable colony was found with nubbins from different clusters and even less from different SSHs and (b) the maximum *N_A_* and *D* observed between two intracolonial MLGs were 13 and 0.37, respectively (i.e., below the modes of the intercluster comparisons). Furthermore, to define the threshold between mosaicism and chimerism, among intracluster comparisons, both MLGs of intracolonial and intercolonial nubbins were compared. These latter comparisons could lead to “artificial” chimerism by virtually fusing nubbins sometimes highly genetically differentiated. Above a certain limit of differentiation, the resulting “artificial” chimera might be unviable (the fusion *in natura* might be impossible or, if remaining possible, might lead to intracolonial conflicts till death of one or all parts of the chimera).

Defining the threshold distinguishing mosaicism and chimerism at four alleles and *D* = 0.12, nine chimeras were detected (9.7% of all colonies). However, by changing this threshold by more or less one allele, the number of chimeras varied to 6 and 14, respectively (i.e., 6.5% and 15.1% of all colonies). Similarly, changing the Bruvo's distance threshold to 0.083 (e.g., four alleles over 24 differing by one mutation step each) or 0.146 (e.g., four alleles over 24 differing by three mutation steps each) would lead to 16 or 7 chimeras, respectively (i.e., 17.2% and 7.5% of all colonies). Schweinsberg et al. (2015) distinguished mosaicism and chimerism from two different ways: colonies were chimeras if nubbins had alleles differing in size from at least (a) 25 bp or (b) four mutation steps. Indeed, according to the authors, such colonies could not be mosaics, as the differences may not come from a single mutation event, nor from various mutation events (the probability that two mutations occurred on the same allele is very low). As some microsatellite loci can mutate without following the SMM (Di Rienzo et al., 1994), defining the threshold between mosaicism and chimerism both from SMM and IAM is expected to be more robust. Puill‐Stephan et al. (2012) considered *A. millepora* newly settled larvae (i.e., recruits) as chimeras when two or more differing alleles were found within nubbins (called subsamples therein) of a single recruit. This latter threshold appears relatively low in the case of adult colonies (in this case, 37.6% of the sampled colonies herein would be chimeras and chimerism would be responsible for 74.5% of IGV). Indeed, during the lifespan of a larva before its settlement, two mutations might rarely occur on two different alleles within the same larva (Puill‐Stephan et al., 2012). This seems more common within an adult colony as time and cellular mitoses allow mutation accumulation.

### 4.3 Mosaicism and chimerism

Most of the genetically variable colonies were identified as mosaics (80.9%). Thus mosaicism appears as the major phenomenon leading to IGV, as already suggested by several studies (e.g., Pineda‐Krch & Lehtila, 2004; Rinkevich, 2004; Santelices, 2004). Of the 93 colonies of *Pocillopora* analyzed in this study (SSH05c‐1: 78; SSH05c‐2: 13; SSH13a: 2), 38 (40.9%) were strictly mosaics (among which six presented three MLGs), revealing that intracolonial mutations are widespread. Almost one‐third of these mosaic colonies presented only one nubbin with an MLG differing from the two others from only one allele, suggesting that a mutation probably appeared, and was maintained in one polyp that then multiplied.

Considering chimerism, about 10% of the analyzed colonies were identified as chimeras. This rate is slightly higher than in Schweinsberg et al. (2015), with the proportion of chimeras ranging from 2.4% to 4.5% for three *Acropora* species, *Pocillopora* spp., and *Porites australiensis*. Nevertheless, the proportion of chimeric colonies found in this study remains low and confirms previous studies that stated chimeras as rarer than mosaics (e.g., Bishop & Sommerfeldt, 1999; Strassmann & Queller, 2004). However, chimerism appears more frequent in recruits as Puill‐Stephan et al. (2012) found it represented 50% of *A. millepora* recruits in experimental conditions. The majority of these chimeras survived only for 2 years (Puill‐Stephan et al., 2012), suggesting that chimerism is not always long‐term viable. Sampling adult colonies should therefore only represent the proportion of those that resisted to the filter of natural selection.

Three chimeras were found bicolor with a color pattern congruent with the genetic differentiation among the intracolonial nubbins. This might suggest that color phenotypes and genotypes are linked. However, Gélin, Fauvelot, et al. (2017) found that colonies sharing the same MLG did not always display the same color. Two bicolor colonies were already observed in *Montipora verrilli/patula* from Hawaii, resulting from either phenotypic plasticity, chimerism, or two adjacent colonies (Johnston, Forsman, & Toonen, 2017). In this study, we considered a colony as a spatially isolated physical entity. As no visible fusion line was obvious within the three bicolor colonies (Figure 4), each appeared to be a single entity and therefore a chimera. Finally, we found some MLGs that were shared among different variable colonies and, interestingly, among different chimeras. These MLGs were already sampled in a previous study (Gélin, Fauvelot, et al., 2017) dealing with clonal propagation among *Pocillopora* SSH05c populations from Reunion Island. In particular, among these MLGs, one (MLG01 in Gélin, Fauvelot, et al., 2017) was previously found overrepresented in REU2 (representing 81% of 264 sampled colonies). The over‐representation of this MLG probably induced its presence within two chimeras (higher sampling probability). However, the two other MLGs shared among different chimeras (MLG06 and MLG19 in Gélin, Fauvelot, et al., 2017) were less represented (23% and 7% of 42 sampled colonies in REU3, respectively; Gélin, Fauvelot, et al., 2017). This suggests that some genetic factors might influence the formation of a chimera as, for example, the fusion between particular MLGs, which would be more viable or more probable than others.

This study attested for the first time the presence of IGV in *Pocillopora* colonies in the southwestern Indian Ocean. The phenomenon appeared widespread in all sampled sites (up to 58%) and mostly resulting from somatic mutations (81%). Nevertheless, chimeras were also found in each site. Based on the high proportions of genetic heterogeneity found, it seems that the benefits provided by IGV overcome the disadvantages for the colony. It is therefore undeniable that it could have ecological and evolutionary implications for which more studies are needed to assess the importance and the role of IGV.

## CONFLICT OF INTEREST

The authors state that there is no conflict of interest.

## AUTHOR CONTRIBUTIONS

NO, PG and HM conceived the idea, designed the experiment, did field and laboratory steps and analyzed the data. NO wrote the original draft, and NO, PG, and HM reviewed and edited the manuscript.

## Data Availability

Data are deposited on Zenodo: https://doi.org/10.5281/zenodo.3490382.
