# Supplementary material for: Together stronger: Intracolonial genetic variability occurrence in Pocillopora corals suggests potential benefits
Source: Ecol Evol. 2020 Jun 5;10(12):5208–18. doi: 10.1002/ece3.5807 (PMC7319244; doi:10.1002/ece3.5807)
Supplement: Supplementary file 1 [file ECE3-10-5208-s001.docx]

**Supporting Information for:**

**Together stronger: intracolonial genetic variability occurrence in *Pocillopora* corals suggests potential benefits**

Nicolas Oury, Pauline Gélin & Hélène Magalon

Correspondence: Nicolas Oury, UMR ENTROPIE, Université de La Réunion, Faculté des Sciences et Technologies, 15 bd René Cassin, CS 92003, 97744 St Denis Cedex 09, La Réunion. Email: [nicolasoury@hotmail.fr](mailto:nicolasoury@hotmail.fr)

**TABLE S1** List of the loci used in this study. Proportions of missing data per locus (*%NA*) are given for all colonies, for each Secondary Species Hypothesis (SSH) and for each cluster separately (number of colonies in parentheses).

| **Panel** | **Locus name** | **Dye** | **Repeat motif** | **Reference** | ***%NA*** | | | | |
| --- | --- | --- | --- | --- | --- | --- | --- | --- | --- |
|  |  |  |  |  | **Total**  **(96)** | **SSH05c** | | | **SSH13a**  **(2)** |
|  |  |  |  |  |  | **Total**  **(94)** | **SSH05c‑1**  **(80)** | **SSH05c‑2**  **(14)** |  |
| 1 | Pd3-004 | 6-FAM | (ATG)_8_ | [4] | 11.8% | 12.1% | 10.8% | 19.0% | 0.0% |
|  | Poc40 | 6-FAM | (CAA)_X_ | [3] | 41.3% | 42.2% | 41.7% | 45.2% | 0.0% |
|  | Pd3-005 | NED | (TGA)_9_ | [4] | 20.8% | 21.3% | 20.0% | 28.6% | 0.0% |
|  | PV2 | VIC | (GA)_20_ | [2] | 20.1% | 18.4% | 17.5% | 23.8% | 100.0% |
|  | PV7 | VIC | (GT)_5_ (CT)_2_ GT (CT)_3_ | [2] | 18.8% | 19.1% | 17.9% | 26.2% | 0.0% |
| 2 | Pd2-001 | VIC | (CA)_11_ | [4] | 23.3% | 23.4% | 22.1% | 31.0% | 16.7% |
|  | Pd2-006 | NED | (CA)_8_ | [4] | 22.6% | 23.0% | 22.1% | 28.6% | 0.0% |
|  | Pd3-008 | 6-FAM | (CTG)_7_ | [4] | 14.9% | 15.2% | 13.3% | 26.2% | 0.0% |
|  | Pd3-009 | 6-FAM | (CAA)_7_ (GAG)_6_ | [4] | 41.7% | 42.2% | 42.1% | 42.9% | 16.7% |
| 3 | Pd3-EF65 | PET | (GTT)_5_ (TGC)_11_ | [1] | 21.2% | 21.6% | 20.0% | 31.0% | 0.0% |
|  | Pd4 | 6-FAM | (AAAC)_5_ | [5] | - | - | - | - | - |
|  | Pd11 | VIC | (CA)_7_ T (AC)_13_ | [5] | 21.9% | 22.3% | 20.8% | 31.0% | 0.0% |
|  | Pd13 | NED | (TCTT)_5_ | [5] | 19.4% | 19.9% | 19.2% | 23.8% | 0.0% |
|  |  |  |  | ***Total*** | ***23.1%*** | ***23.4%*** | ***22.3%*** | ***29.8%*** | ***11.1%*** |

[1] Gorospe, K. D., & Karl, S. A. (2013). Genetic relatedness does not retain spatial pattern across multiple spatial scales: dispersal and colonization in the coral, *Pocillopora damicornis*. *Molecular Ecology*, *22*, 3721–3736. https://doi.org/10.1111/mec.12335

[2] Magalon, H., Samadi, S., Richard, M., Adjeroud, M., & Veuille, M. (2004). Development of coral and zooxanthella-specific microsatellites in three species of *Pocillopora* (Cnidaria, Scleractinia) from French Polynesia. *Molecular Ecology Notes*, *4*, 206–208. https://doi.org/10.1111/j.1471-8286.2004.00618.x

[3] Pinzón, J. H., & LaJeunesse, T. C. (2011). Species delimitation of common reef corals in the genus *Pocillopora* using nucleotide sequence phylogenies, population genetics and symbiosis ecology. *Molecular Ecology*, *20*, 311–325. https://doi.org/10.1111/j.1365-294X.2010.04939.x

[4] Starger, C. J., Yeoh, S. S., Dai, C.-F., Baker, A. C., & Desalle, R. O. B. (2008). Ten polymorphic STR loci in the cosmopolitan reef coral, *Pocillopora damicornis*. *Molecular Ecology Resources*, *8*, 619–621. https://doi.org/10.1111/j.1471-8286.2007.02017.x

[5] Torda, G., Schmidt-Roach, S., Peplow, L. M., Lundgren, P., & van Oppen, M. J. H. (2013). A rapid genetic assay for the identification of the most common *Pocillopora damicornis* genetic lineages on the great barrier reef. *PLoS ONE*, *8*, e58447. https://doi.org/10.1371/journal.pone.0058447

**TABLE S2** Summary of the genetic information about chimeras. Multilocus genotypes (MLGs) are given for each nubbin (noted a, b and c within each colony). Non amplified loci are represented by “?” and intracolonial differing alleles are in bold. Correspondence of MLGs without missing data with those of Gélin, Fauvelot, et al. (2017) is given in the “MLG” column. *N_L_* and *N_A_* are respectively the number of comparable loci and the number of different alleles between two intracolonial nubbins, *D* is the Bruvo’s distance (Bruvo, Michiels, D’Souza, & Schulenburg, 2004).

| **Colony** | **Cluster** | **Nubbin** | **MLG** | **Pd2-001** | **Pd3-004** | **Pd3-005** | **Pd2-006** | **Pd3-008** | **Pd3-009** | **PV2** | **PV7** | **Poc40** | **Pd11** | **Pd13** | **Pd3-EF65** | **Comparison** | ***N_L_*** | ***N_A_*** | ***D*** |
| --- | --- | --- | --- | --- | --- | --- | --- | --- | --- | --- | --- | --- | --- | --- | --- | --- | --- | --- | --- |
| C1  (REU2) | SSH05  c-1 | a | MLG01 | 218-218 | 177-177 | 231-231 | 211-211 | 185-**185** | 337-346 | 147-181 | 248-248 | **320-320** | **172-172** | 164-164 | 206-215 | a-b | 10 | 5 | 0.14 |
|  |  | b |  | 218-218 | 177-177 | 231-231 | ? | 185-**191** | ? | 147-181 | 248-248 | **314-314** | **170-170** | 164-164 | 206-215 | b-c | 2 | 2 | 0.05 |
|  |  | c |  | ? | **180-183** | 231-231 | ? | ? | ? | ? | ? | ? | ? | ? | ? | a-c | 2 | 2 | 0.05 |
| C2  (REU3) | SSH05  c-1 | a |  | ? | **174-174** | ? | ? | **188-188** | ? | 147-**147** | ? | ? | ? | ? | ? | a-b | 3 | 5 | 0.13 |
|  |  | b |  | 218-218 | **177-177** | 231-231 | 211-211 | 185-185 | ? | 147-**181** | 248-248 | 320-320 | 172-172 | 164-164 | 206-215 | b-c | 3 | 2 | 0.07 |
|  |  | c |  | ? | **168-168** | ? | ? | 185-185 | ? | ? | ? | ? | ? | ? | 206-215 | a-c | 2 | 4 | 0.10 |
| C3  (REU3) | SSH05  c-1 | a | MLG06 | 218-218 | 177-**180** | 228-**231** | **211**-213 | **182**-185 | 358-**361** | 147-147 | 248-248 | **314-320** | **164-164** | 164-188 | **215-215** | a-b | 12 | 11 | 0.31 |
|  |  | b | MLG19 | 218-218 | 177-**177** | 228-**228** | **213**-213 | 185-185 | 358-**358** | 147-147 | 248-248 | **323-323** | **172-172** | 164-188 | **206-209** | b-c | 2 | 3 | 0.08 |
|  |  | c |  | ? | **171-171** | ? | ? | 185-**188** | ? | ? | ? | ? | ? | ? | ? | a-c | 2 | 4 | 0.11 |
| C4  (REU3) | SSH05  c-1 | a |  | 216-216 | 177-177 | 231-240 | 211-211 | 185-185 | ? | 147-181 | 248-248 | 323-323 | 164-172 | 164-184 | 206-215 | a-b | 11 | 1 | 0.04 |
|  |  | b |  | 216-216 | 177-177 | 231-240 | 211-211 | 185-185 | 337-**337** | 147-**147** | 248-248 | 323-323 | 164-172 | 164-184 | 206-215 | b-c | 12 | 9 | 0.28 |
|  |  | c | MLG01 | **218-218** | 177-177 | 231-**231** | 211-211 | 185-185 | 337-**346** | 147-181 | 248-248 | **320-320** | **172**-172 | 164-**164** | 206-215 | a-c | 11 | 7 | 0.20 |
| C5  (REU3) | SSH05  c-1 | a | MLG72 | 218-218 | 177-177 | 228-228 | 213-213 | 185-185 | 358-358 | 147-147 | 248-248 | **317**-323 | 172-172 | 164-188 | 206-209 | a-b | 12 | 1 | 0.03 |
|  |  | b | MLG19 | 218-218 | 177-177 | 228-228 | 213-213 | 185-185 | 358-358 | 147-147 | 248-248 | **323**-323 | 172-172 | 164-188 | 206-209 | b-c | 11 | 10 | 0.29 |
|  |  | c |  | 218-218 | 177-**180** | 228-**231** | **211**-213 | **182**-185 | ? | 147-147 | 248-248 | **314-320** | **164-164** | 164-188 | **215-215** | a-c | 11 | 10 | 0.27 |
| C6  (REU3) | SSH05  c-2 | a |  | 218-218 | 177-**177** | 228-**228** | **213**-213 | **185**-185 | 358-**358** | 147-147 | 248-248 | **317-317** | **172-172** | 164-188 | **206-209** | a-b | 12 | 11 | 0.29 |
|  |  | b | MLG06 | 218-218 | 177-180 | 228-231 | 211-213 | 182-185 | 358-**361** | 147-147 | 248-248 | 314-320 | **164-164** | 164-188 | **215-215** | b-c | 8 | 1 | 0.04 |
|  |  | c |  | ? | 177-180 | 228-231 | 211-213 | 182-185 | ? | 147-147 | 248-248 | 314-320 | ? | 164-**164** | ? | a-c | 8 | 7 | 0.17 |
| C7  (REU3) | SSH05  c-1 | a |  | 216-216 | 177-177 | 231-240 | 211-211 | 185-185 | ? | 147-**147** | 248-248 | 323-323 | 164-172 | 164-184 | 206-215 | a-b | 11 | 9 | 0.26 |
|  |  | b | MLG02 | **218-218** | 177-177 | **228-231** | 211-211 | 185-185 | 337-**346** | **181**-181 | 248-248 | **320-320** | 164-172 | 164-**164** | 206-215 | b-c | 12 | 9 | 0.26 |
|  |  | c | MLG03 | 216-216 | 177-177 | 231-240 | 211-211 | 185-185 | 337-**337** | 147-181 | 248-248 | 323-323 | 164-172 | 164-184 | 206-215 | a-c | 11 | 1 | 0.04 |
| C8  (REU4) | SSH05  c-1 | a |  | 216-216 | 177-177 | 231-240 | 211-211 | 185-185 | 337-337 | 147-181 | 248-248 | ? | 164-172 | 164-184 | 206-215 | a-b | 11 | 0 | 0.00 |
|  |  | b |  | 216-216 | 177-177 | 231-240 | 211-211 | 185-185 | 337-337 | 147-181 | 248-248 | ? | 164-172 | 164-184 | 206-215 | b-c | 8 | 5 | 0.12 |
|  |  | c |  | ? | 177-177 | 231-240 | 211-211 | **184-184** | ? | 147-**147** | ? | ? | **170-170** | 164-184 | 206-215 | a-c | 8 | 5 | 0.12 |
| C9  (REU4) | SSH05  c-2 | a |  | 218-218 | 177-177 | 231-249 | 211-213 | 182-182 | 361-361 | 147-181 | 248-248 | ? | 164-170 | 192-192 | 215-218 | a-b | 11 | 0 | 0.00 |
|  |  | b |  | 218-218 | 177-177 | 231-249 | 211-213 | 182-182 | 361-361 | 147-181 | 248-248 | ? | 164-170 | 192-192 | 215-218 | b-c | 11 | 13 | 0.37 |
|  |  | c |  | **216-216** | 177-177 | 231-**240** | 211-**211** | **185-185** | **337-337** | 147-181 | 248-248 | ? | 164-**172** | **164-184** | **206-215** | a-c | 11 | 13 | 0.37 |

Bruvo, R., Michiels, N. K., D’Souza, T. G., & Schulenburg, H. (2004). A simple method for the calculation of microsatellite genotype distances irrespective of ploidy level. *Molecular Ecology, 13*, 2101–2106. https://doi.org/10.1111/j.1365-294X.2004.02209.x

Gélin, P., Fauvelot, C., Mehn, V., Bureau, S., Rouzé, H., & Magalon, H. (2017). Superclone expansion, long-distance clonal dispersal and local genetic structuring in the coral *Pocillopora damicornis* type *β* in Reunion Island, South Western Indian Ocean. *PLoS ONE*, *12*, e0169692. https://doi.org/10.1371/journal.pone.0169692

**FIGURE S1** Threshold of the number of comparable loci between two intracolonial nubbins (*N_L_*). The histogram (left axis) represents the distribution of the number of comparable loci between two intracolonial nubbins (*N_L_*) within invariable colonies. For each *N_L_*, the “false negative” probability (i.e., the probability of detecting a colony as invariable while it is actually *variable*) is estimated (brown continuous line; right axis). The dashed line symbolized the arbitrary threshold of *N_L_* distinguishing colonies *invariable* from those *possibly variable*.

**FIGURE S2** Proportions of the categories of genetic variability per site (indicated above) for each SSH05c cluster and each Secondary Species Hypothesis (SSH) separately (number of colonies in parentheses below). The hatched parts correspond to colonies for which at least one intracolonial comparison was done with less than nine loci (*N_L_* < 9).


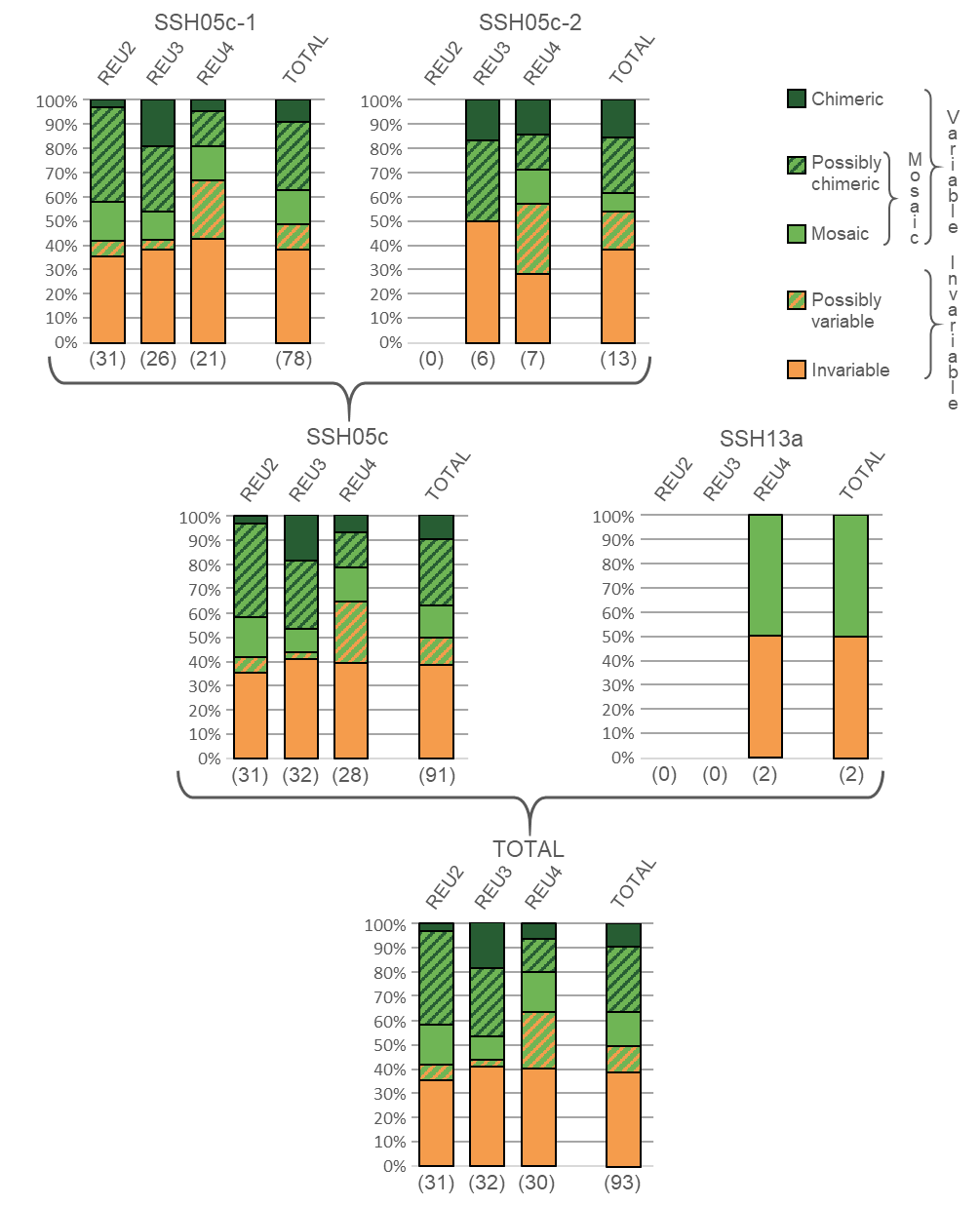


**FIGURE S3** Thresholds between mosaicism and chimerism at 11 loci. (a) distribution of the number of different alleles (*N_A_*) between two multilocus genotypes (MLGs) and (b) distribution of the Bruvo’s distance (*D*; Bruvo, Michiels, D’Souza, & Schulenburg, 2004) between two MLGs. Only MLGs without missing data were compared by pair (*N* = 7,140 paired comparisons, including 4,987 intra-cluster, 1,683 inter-cluster and 464 inter-SSH comparisons). The categories of genetic variability are indicated above each chart. *N_A_*_CHI/MOS_ and *D*_CHI/MOS_ are the genetic differentiation thresholds between mosaicism and chimerism.


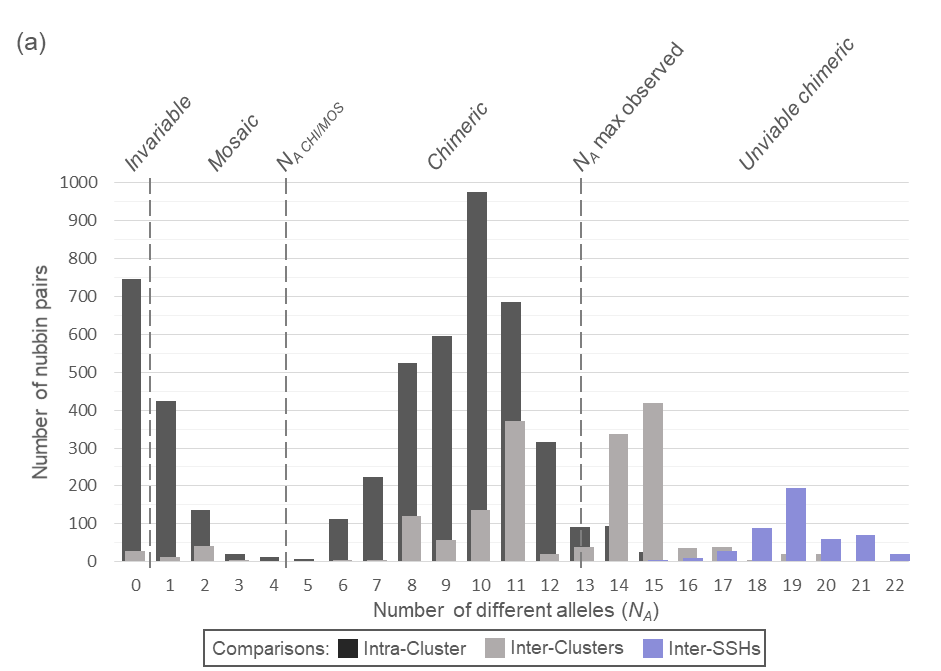


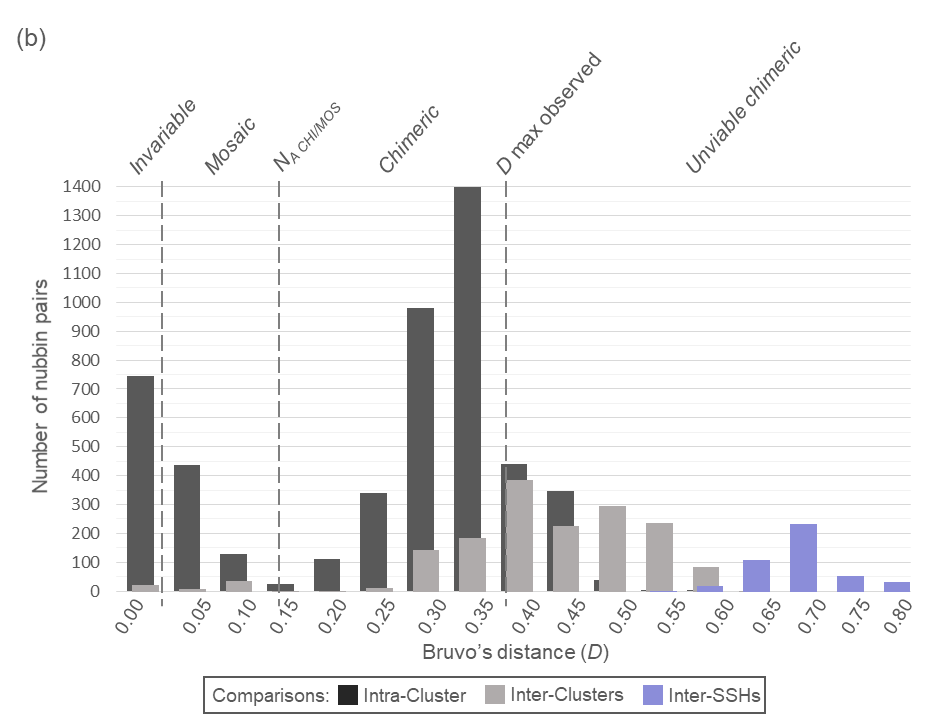


Bruvo, R., Michiels, N. K., D’Souza, T. G., & Schulenburg, H. (2004). A simple method for the calculation of microsatellite genotype distances irrespective of ploidy level. *Molecular Ecology, 13*, 2101–2106. https://doi.org/10.1111/j.1365-294X.2004.02209.x
